# Autism Spectrum Disorder and Atypical Epilepsy Presentation in KCNQ3 Mutations: Expansion of Phenotypic Spectrum With Neuroimaging Findings

**DOI:** 10.1155/crpe/4543154

**Published:** 2026-05-31

**Authors:** Danilo de Assis Pereira, Rafael Saliba Helmer, Matheus Luis de Souza Silva, Lisiane Seguti Ferreira

**Affiliations:** ^1^ Department of Human Reproduction and Pediatrics, Faculty of Medical Sciences of Sorocaba, Pontifical Catholic University, Sorocaba, São Paulo, Brazil, puc-rio.br; ^2^ State University of Campinas, Campinas, São Paulo, Brazil, unicamp.br; ^3^ Pediatric Neuroradiology Service, Diagnósticos da América SA Private Practice, Barueri, Brazil; ^4^ Faculty of Medicine, University of Brasília, Federal District, Brasília, Brazil, unb.br

**Keywords:** autism spectrum disorder, channelopathy, developmental encephalopathy, epilepsy, KCNQ3, neuroimaging

## Abstract

**Background:**

Mutations in the KCNQ3 gene are primarily associated with benign familial neonatal epilepsy; however, recent studies have expanded its phenotypic spectrum to include developmental and epileptic encephalopathies (DEE) and neurodevelopmental disorders, including autism spectrum disorder (ASD). This report describes an atypical presentation of a patient with a de novo KCNQ3 mutation, manifesting as neonatal‐onset epilepsy, ASD traits, and abnormal neuroimaging findings, contributing to the evolving understanding of KCNQ3‐related disorders.

**Case Presentation:**

A 5‐year‐old boy presented with neonatal seizures that initially responded to antiepileptic therapy but subsequently relapsed with atypical, drug‐resistant seizures. Neurodevelopmental assessment revealed moderate intellectual disability, ASD features, and speech delay. Brain MRI showed hippocampal asymmetry and diffuse enlargement of perivascular spaces, raising questions about potential structural correlates of genetic epilepsies. Whole‐exome sequencing identified a de novo heterozygous KCNQ3 variant, absent in both parents.

**Discussion:**

This case highlights the expanding phenotypic variability of KCNQ3 mutations, supporting their role in epileptic encephalopathies and neurodevelopmental disorders. While previous reports primarily associate KCNQ3 mutations with early‐onset epilepsy, this case suggests a broader neurodevelopmental impact, including ASD traits and neuroimaging abnormalities, emphasizing the importance of genetic screening in complex epilepsy syndromes. Additionally, the hippocampal asymmetry and prominent perivascular spaces warrant further investigation into their relevance in KCNQ3‐related disorders.

**Conclusion:**

This study expands the clinical and neuroimaging spectrum of KCNQ3‐related epileptic encephalopathy, reinforcing its association with neurodevelopmental comorbidities. Early genetic diagnosis may guide treatment choices and provide valuable prognostic insights, advocating for a multidisciplinary approach in managing these patients.

## 1. Case Report

We present the case of a six‐year‐old female patient, born to nonconsanguineous parents (a 28‐year‐old mother and a 30‐year‐old father) following an uneventful pregnancy. Delivery occurred at full term (39 weeks) via elective cesarean section, with a birth weight of 3100 g and Apgar scores of 8 and 9 at the first and fifth minutes, respectively.

### 1.1. Epilepsy

At 2 days of life, the neonate exhibited the first episodes of paroxysmal distress, characterized by a fixed gaze, perioral cyanosis, and transient generalized rigidity, consistent with neonatal seizures. Approximately four similar episodes occurred within a 24‐h period, necessitating admission to a neonatal intensive care unit. A neonatal electroencephalogram (EEG) revealed a burst‐suppression pattern, prompting the initiation of intravenous phenobarbital, which effectively controlled the seizures. The patient was discharged at 20 days of life while on oral phenobarbital.

For several months postdischarge, the patient remained seizure free. Phenobarbital was gradually tapered off at 6 months of age. However, at 8 months, she experienced a febrile seizure, a three‐minute bilateral tonic–clonic seizure during an upper respiratory infection. Between 8 months and 2 years of age, no afebrile seizures were reported. At 2 years and 3 months, spontaneous generalized tonic–clonic seizures emerged, initially infrequent (one to two per month) but progressively increasing in frequency, eventually clustering in series. By 3 years of age, she was experiencing multiple daily seizures, with some progressing to status epilepticus, necessitating several hospitalizations. Treatment involved multiple combinations of antiepileptic drugs, including valproate, topiramate, and clobazam, as well as a ketogenic diet, which was initiated at 4 years of age. The introduction of the ketogenic diet at 4 years of age led to a partial reduction in seizure frequency, though without complete remission, suggesting a moderate therapeutic effect.

Currently, at 6 years old, she is maintained on valproic acid and clobazam, with a significant reduction in seizure frequency (the last episode occurring 4 months prior).

### 1.2. Physical Examination and Developmental Evaluation

Developmental milestones, both motor and linguistic, were markedly delayed. The patient achieved head control at 6 months, attained independent sitting at 12 months, and was only able to walk unassisted after the age of 3 years. At 6 years old, she remains unable to formulate sentences and can articulate only a limited number of isolated words. Cognitively and socially, the patient exhibits behavioral features consistent with a formal diagnosis of autism spectrum disorder (ASD) according to DSM‐5 criteria, classified as Level 2 (requiring substantial support). These include persistent deficits in social communication, reduced eye contact, limited symbolic play, and repetitive behavioral patterns. Importantly, the autistic features are not secondary to intellectual disability, as they were evident independently of global cognitive performance. A comprehensive neuropsychological assessment at age five confirmed moderate intellectual disability with an estimated IQ of approximately 50.

The patient exhibited physical findings, including slightly enlarged auricular helices, ocular hypertelorism with epicanthal folds, a broad and low‐set fontanelle, maxillary hypoplasia, a thin lower lip, and a short neck. Her fingers were fusiform and lacked palmar creases. Pulmonary and cardiac auscultation revealed no significant abnormalities.

Neurological examination revealed strabismus, mild hypertonia in the lower limbs and diffuse hyperreflexia, while muscle strength was preserved, and no cerebellar signs were observed. An ophthalmological evaluation confirmed the presence of strabismus and dyschromatopsia.

### 1.3. Complementary Examinations

Complementary assessments included a karyotype (46, XX [20]) that was within normal limits, a urinary system ultrasound without abnormalities, a cranial computed tomography (CT) scan, audiometry and brainstem auditory evoked potential, with no significant findings, and a normal metabolic screening panel. Laboratory tests, including hepatic enzymes (AST, ALT, and GGT), ammonia, lactate, free T4, TSH, urea, and creatinine, were within normal reference ranges. A lipid profile showed hypercholesterolemia and hypertriglyceridemia, while the bronchial provocation test confirmed the presence of bronchial hyperreactivity.

### 1.4. Magnetic Resonance Imaging (MRI) Findings

**FIGURE 1:**
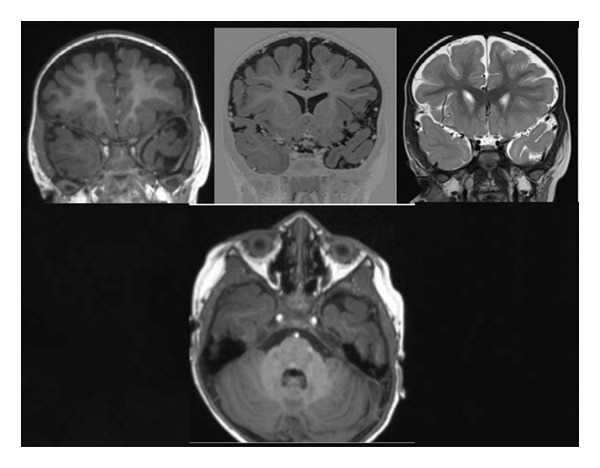
Coronal T1‐ and T2‐weighted MRI sequences demonstrating asymmetry of the temporal lobes. The left temporal lobe and hippocampus exhibit a mild volume reduction compared with the right side.

**FIGURE 2:**
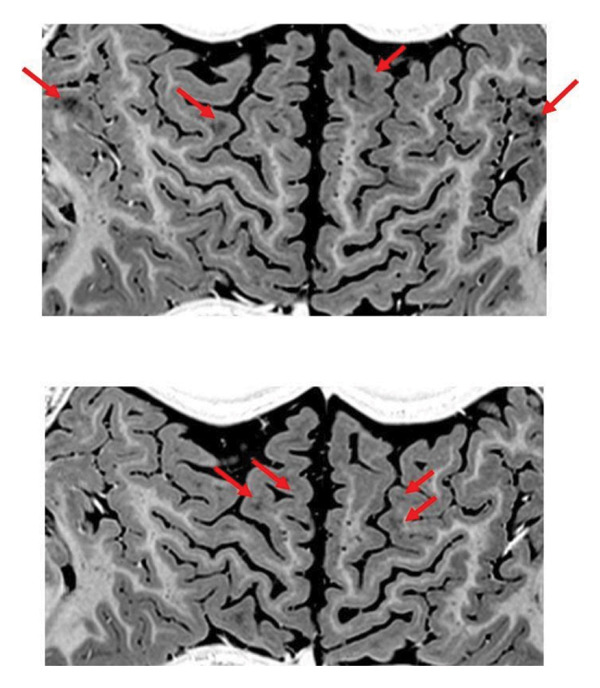
Axial T1‐weighted MRI sequence revealing multiple enlarged perivascular spaces (Virchow–Robin spaces), appearing as punctate hyperintense areas scattered throughout the subcortical white matter. These cystic perivascular dilations (examples indicated by red arrows) are particularly prominent in the bilateral frontal regions. There is no associated edema, mass effect, or abnormal contrast enhancement.

**FIGURE 3:**
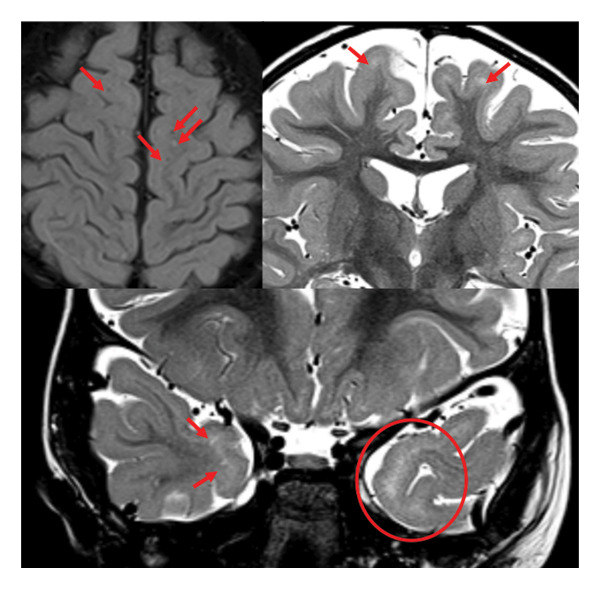
Axial FLAIR (top left) and coronal T2‐weighted (top right and bottom) MRI sequences demonstrating scattered enlargement of perivascular spaces in the subcortical regions (red arrows).

Brain MRI performed at 5 years of age revealed subtle abnormalities in the cerebral white matter, as well as enlarged perivascular spaces, predominantly in the bilateral frontoparietal regions (Figures 1, 2, and 3). No major structural malformations or gross volumetric asymmetries were identified.

### 1.5. EEG Findings at Two Years

During both EEG recordings, performed during non‐REM sleep under good technical conditions, background activity was composed of medium‐amplitude, irregular slow waves in the theta–delta range. In the first exam (September 18, 2024), sleep graph elements such as vertex sharp waves and sleep spindles were reduced for age, and there was a mild disturbance in background organization. In the second exam (January 29, 2025), sleep architecture was more organized, with normal age‐appropriate graph elements, including K‐complexes.

**FIGURE 4:**
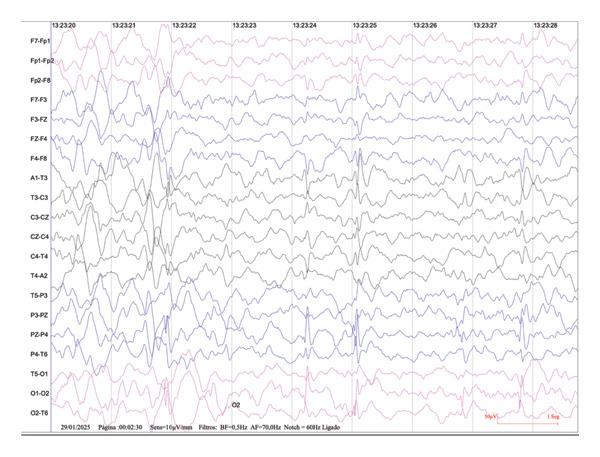
EEG recorded during NREM sleep showing bilateral parieto‐occipital sharp waves, consistent with epileptiform activity in a patient with a de novo KCNQ3 (p.Arg230His) variant.

In both recordings, frequent epileptiform discharges were observed over the parietal and occipital regions bilaterally (Figure 4). These discharges were characterized by medium‐amplitude sharp waves, occurring both synchronously and asynchronously, sometimes in brief trains. In the second recording, there were also occasional sharp waves in the right frontal region, with propagation to adjacent areas. Phase reversals were noted in electrodes P4, O2, P3, O1, and F8.

### 1.6. Genetic Diagnosis

A whole‐exome sequencing identified a de novo pathogenic variant, KCNQ3: NM_004519.4:c.689G > A:p. (Arg230His), in heterozygosity (46.9%) in exon 4 of the KCNQ3 gene. This missense variant results in a nucleotide substitution leading to an amino acid change in the encoded protein.

## 2. Discussion

### 2.1. Genetic and Phenotypic Spectrum of KCNQ2/3 Channelopathies

Ion channelopathies involving mutations in the KCNQ gene family represent a clinical continuum that ranges from self‐limited (previously referred to as “benign”) neonatal epilepsies to severe developmental and epileptic encephalopathies (DEE) [1]. The first familial neonatal seizure syndrome linked to KCNQ2 was described in 1998, and subsequent discoveries identified KCNQ3 as another key gene implicated in these phenotypes [2]. Although initial cases were associated with favorable outcomes, subsequent reports have expanded the phenotypic spectrum to include more severe epileptic and developmental outcomes.

In the present case, the patient exhibited early‐onset seizures during the neonatal period, initially suggestive of a KCNQ3‐related familial epilepsy. However, electroencephalographic findings revealed a burst‐suppression pattern, a hallmark of epileptic encephalopathy and inconsistent with a typical self‐limited presentation. Furthermore, seizure recurrence and clinical deterioration after the neonatal phase, accompanied by significant developmental delay, fulfilled criteria for DEE [3].

This clinical trajectory is consistent with previously reported sporadic cases involving de novo mutations in KCNQ3, in which seizures persist or re‐emerge beyond the neonatal period and are often associated with cognitive impairment [4, 5]. Nearly all known pathogenic KCNQ3 variants implicated in DEE are heterozygous, exhibit autosomal dominant inheritance, and most commonly arise de novo, with unaffected parents [5]. For instance, Allen et al. described de novo KCNQ3 mutations in infants presenting with early‐onset epileptic encephalopathy [5]. In line with this pattern, the KCNQ3 variant identified in our patient was absent in both parents, and family history was negative for epilepsy.

A distinguishing feature of KCNQ3‐related disorders is their marked phenotypic heterogeneity (see Table 1) [5, 6]. In addition to neonatal seizures and global developmental delay observed in this case, other individuals harboring identical KCNQ3 mutations have exhibited diverse clinical manifestations. Arredondo et al. described a family carrying a heterozygous p.R364H KCNQ3 variant with phenotypes ranging from ASD and anxiety to childhood absence epilepsy and adult‐onset epilepsy [6].

**TABLE 1:** Comparison of KCNQ3 mutations and their clinical phenotypes.

Mutation	Variant type	Functional impact	Epilepsy	Cognitive delay	Autism	Other findings	Neuroimaging findings
p.R213Q	Missense	Loss‐of‐function	Yes	Moderate	Yes	Neonatal hypotonia	Normal MRI
p.A315T	Missense	Gain‐of‐function	Yes	Mild	No	Stereotyped movements	Not reported
p.T326M	Missense	Loss‐of‐function	Yes	Severe	Yes	White matter abnormalities	Diffuse white matter signal changes
p.R364H	Missense	Loss‐of‐function	Yes	Mild–Moderate	Yes	Childhood absence epilepsy	Mild cerebral atrophy
p.G256V	Missense	Indeterminate	Yes	Mild	Yes	Speech delay	Normal MRI
Patient in this report	Missense	Likely loss‐of‐function	Neonatal seizures with burst suppression; relapse postnatally	Severe	Yes	Hypotonia, delayed milestones	Global cerebral atrophy, hypomyelination

KCNQ3 pathogenic variants are classically associated with benign familial neonatal seizures type 2 (BFNS2; OMIM: 121201), characterized by brief (1‐2 min) tonic, focal clonic, or apneic seizures accompanied by autonomic features, with onset between days 2 and 8 of life, and spontaneous resolution by 6–12 months. These patients typically demonstrate normal psychomotor development. However, other KCNQ3‐related phenotypes include neurodevelopmental disorders marked by intellectual disability, with or without epilepsy or cortical visual impairment. Importantly, due to the limited number of documented cases, the full clinical spectrum of KCNQ3‐associated neurodevelopmental disorders (KCNQ3‐NDD) remains incompletely defined [2, 4].

This case highlights the importance of genetic testing and precise electroclinical correlation, particularly when the clinical course deviates from the expected trajectory of self‐limited epilepsies. The presence of burst suppression, seizure persistence, and developmental impairment supports classification within the DEE spectrum, reinforcing the phenotypic variability and diagnostic complexity of KCNQ3‐related channelopathies.

### 2.2. Comparison of KCNQ3 Mutations and Their Clinical Phenotypes

Table 1 provides an expanded overview of various KCNQ3 mutations and their associated clinical phenotypes, emphasizing not only the diversity in functional impact and neurodevelopmental outcomes but also the role of neuroimaging findings, which remain underreported in the literature. The inclusion of neuroimaging data is essential to understanding the broader structural brain alterations potentially linked to Kv7.3 dysfunction.

Most ASD‐associated KCNQ3 variants result in loss‐of‐function, leading to neuronal hyperexcitability and impaired Kv7.3‐mediated potassium currents [5, 7]. Variants associated with childhood absence epilepsy (e.g., p.R364H) typically correlate with milder cognitive phenotypes, whereas those linked to early‐onset DEE often present with severe intellectual disability, motor deficits, and cortical atrophy. Recent genotype–phenotype correlations have identified p.T326M and p.R213Q variants in individuals with ASD, further suggesting a mechanistic overlap between epileptic and neurodevelopmental disorders [4, 7].

In the present case, the patient exhibited seizures within the first week of life, initially raising suspicion for a familial KCNQ3‐related neonatal epilepsy. However, video‐EEG monitoring revealed a burst‐suppression pattern, consisting of alternating high‐voltage bursts and 5–10 s suppression periods, a hallmark of Ohtahara syndrome, and other early epileptic encephalopathies. Over time, seizures relapsed during infancy and evolved into multifocal and generalized tonic and tonic–clonic seizures, often triggered by infections or sleep deprivation. These were associated with marked neurodevelopmental regression, including hypotonia, loss of eye contact, and loss of previously acquired motor milestones.

Neuroimaging findings included diffuse cerebral atrophy, hypomyelination, and prominent extra‐axial spaces, particularly in the frontal lobes. As the number of reported cases increases, future efforts should prioritize systematic neuroimaging characterization, which may uncover genotype‐specific structural signatures and improve early diagnostic stratification.

### 2.3. Mechanisms Linking KCNQ3 Mutations and ASD

Mechanisms linking KCNQ3 mutations and ASD include impaired Kv7‐mediated neuronal inhibition, reducing M‐current activity, leading to hyperexcitability and disruption of critical developmental networks [7]; impact on GABAergic circuits, as shown by studies in mouse models with KCNQ3 dysfunction revealing abnormalities in inhibitory neurotransmission, a mechanism previously implicated in ASD pathophysiology [5, 8]; and developmental disruptions, associated with speech delay, intellectual disability, and repetitive behaviors, core features of ASD [5]. Lauritano et al. reported a KCNQ3 variant (p.R213Q) in a cohort of children with epilepsy and ASD traits, supporting the hypothesis that KCNQ3 dysfunction may contribute to the broader spectrum of neurodevelopmental disorders [4]. Our patient exhibited moderate intellectual disability, repetitive behaviors, and deficits in social interaction, further reinforcing this association [4, 5].

Clinical implications involve early genetic screening for KCNQ3 mutations in individuals presenting with ASD and epilepsy, potentially improving diagnostic precision and allowing targeted interventions, particularly therapies aimed at modulating Kv7 channel activity [4, 5, 9].

The patient fulfilled DSM‐5 diagnostic criteria for ASD, classified as Level 2. Social‐communication deficits, reduced reciprocity, and restricted behavioral patterns were evident and could not be attributed to the co‐occurring intellectual disability, which was characterized as moderate (IQ≈50). These findings indicate that the autistic phenotype represents an independent neurodevelopmental dimension rather than a secondary consequence of global cognitive impairment.

### 2.4. Neuroimaging Findings in KCNQ‐Associated Epilepsies

Neuroimaging in genetic epilepsies caused by ion channel mutations typically yields normal or nonspecific findings, as the underlying dysfunction is biochemical rather than structural [10]. Indeed, benign KCNQ3‐associated epilepsies usually exhibit normal brain MRI [11, 12]. In severe cases, transient white matter abnormalities or suspected cortical dysplasia have been noted but not consistently confirmed later [10]. Currently, no specific neuroimaging pattern has been established for KCNQ3‐related DEE [2, 4, 9]. Most severe cases reported have shown no fixed structural brain malformations though indirect signs of cerebral injury or maturational delay may be detectable [1, 2, 10]. In our case, the temporal hippocampal asymmetry and diffuse enlargement of perivascular spaces may reflect subtle impacts of epilepsy and genetic disorder on cerebral development [10, 13]. The smaller left hippocampus might result from more intense epileptiform activity in that hemisphere since the neonatal period, leading to subtle atrophy or impaired development [13]. Meanwhile, the diffusely enlarged perivascular (Virchow–Robin) spaces suggest microstructural changes in white matter, possibly related to disruptions in myelination or diffuse neuronal loss though they might also represent a constitutional feature accentuated by mild cerebral atrophy [10, 13]. The neuroimaging findings in this patient, although subtle, contribute significantly to the etiological reasoning. The absence of destructive or focal structural lesions, combined with the presence of diffuse white matter changes and prominent perivascular spaces, supports a nonacquired, potentially genetic etiology. Similar features have been described in patients with channelopathies and early‐onset DEE [13]. Future studies involving quantitative volumetric and microstructural analysis, including assessment of perivascular space burden, may provide further insight into the neuroanatomical correlates of genetic epilepsies.

Advanced MRI techniques, such as diffusion tensor imaging (DTI) and volumetric analyses, could provide further insights into structural and functional connectivity alterations in KCNQ3‐related disorders [10].

The structural abnormalities identified on MRI should be regarded as tentative observations, as no causal relationship with the KCNQ3 variant can be established. The imaging data are descriptive in nature and lack quantitative or microstructural analyses that would allow inference regarding genotype–phenotype coupling.

### 2.5. Therapeutic Implications and Future Directions

Identifying a KCNQ3 mutation has important therapeutic implications. Mutations in these channels can lead to either loss‐of‐function, reducing potassium currents and enhancing neuronal excitability, or, less commonly, gain‐of‐function [14]. For loss‐of‐function mutations, therapies enhancing Kv7 channel activity, such as retigabine (ezogabine), have shown variable success in some KCNQ2 encephalopathy patients [9]. Although retigabine is not readily available clinically, similar targeted treatments might be explored for KCNQ3 mutations in the future [12]. Moreover, understanding the genetic defect avoids the use of potentially less effective medications. Stabilizing neuronal membrane drugs, such as valproate, benzodiazepines, and phenytoin, might be rational choices, whereas medications with different mechanisms might not impact underlying pathophysiology directly [8]. In our patient, valproate and clobazam effectively controlled seizures initially.

The clinical implications of confirming the diagnosis extend beyond immediate seizure management. Recognizing a genetic encephalopathy allows more accurate prognostic discussions, earlier therapeutic planning, and realistic family counseling [7]. Although moderate intellectual disability is expected to persist, multidisciplinary interventions, including speech therapy, occupational therapy, and specialized educational support, may optimize functional outcomes [15]. Clarifying the etiology also helps reduce parental burden by demonstrating that the mutation occurred spontaneously, supporting appropriate genetic counseling [16]. Documenting such cases contributes to a more precise delineation of the clinical and radiological spectrum associated with KCNQ3 variants and strengthens the rationale for systematic genetic evaluation in early‐onset epilepsy. In this context, this case illustrates the importance of considering KCNQ3 mutations in children with early‐onset epilepsy and ASD features, even when the neonatal presentation is atypical, as early genetic identification can refine management, improve prognostic accuracy, and prevent diagnostic delay.

## Funding

The authors have nothing to report.

## Ethics Statement

Written informed consent was obtained from the patient’s mother for the publication of this case report, including clinical details and neuroimaging findings. All ethical considerations were followed in accordance with the Declaration of Helsinki.

## Conflicts of Interest

The authors declare no conflicts of interest.

## Data Availability

Data supporting the findings of this case report are available from the corresponding author upon reasonable request. Due to patient confidentiality and ethical considerations, data will only be shared with qualified researchers upon approval.

## References

[1] Portale A. , Comella M. , Salomone G. et al., The Spectrum of KCNQ2- and KCNQ3-Related Epilepsy, Journal of Pediatric Neurology. (2021) 21, no. 03, 203–211, 10.1055/s-0041-1727099.

[2] Miceli F. , Striano P. , Soldovieri M. V. et al., A Novel KCNQ3 Mutation in Familial Epilepsy With Focal Seizures and Intellectual Disability, Epilepsia. (2015) 56, no. 2, e15–e20, 10.1111/epi.12887, 2-s2.0-84923359250. 25524373

[3] Arredondo K. , Myers C. , Hansen-Kiss E. et al., Phenotypic Spectrum in a Family Sharing a Heterozygous, Journal of Child Neurology. (2022) 37, no. 6, 517–523, 10.1177/08830738221089741. 35384780

[4] Lauritano A. , Moutton S. , Longobardi E. et al., A Novel Homozygous KCNQ3 Loss-of-Function Variant Causes Non-Syndromic Intellectual Disability and Neonatal-Onset Pharmacodependent Epilepsy, Epilepsia Open. (2019) 4, no. 3, 464–475, 10.1002/epi4.12353, 2-s2.0-85070676275. 31440727 PMC6698674

[5] Sands T. T. , Miceli F. , Lesca G. et al., Autism and Developmental Disability Caused by KCNQ3 Gain-of-Function Variants, Annals of Neurology. (2019) 86, no. 2, 181–192, 10.1002/ana.25522, 2-s2.0-85068128686. 31177578

[6] Wang J. , Lin Z. J. , Liu L. et al., Epilepsy-Associated Genes, Seizure. (2017) 44, 11–20, 10.1016/j.seizure.2016.11.030, 2-s2.0-85008354935. 28007376

[7] Neubauer B. A. , Waldegger S. , Heinzinger J. et al., KCNQ2 and KCNQ3 Mutations Contribute to Different Idiopathic Epilepsy Syndromes, Neurology. (2008) 71, no. 3, 177–183, 10.1212/01.wnl.0000317090.92185.ec, 2-s2.0-48449092930. 18625963

[8] Singh N. A. , Otto J. F. , Dahle E. J. et al., Mouse Models of Human KCNQ2 and KCNQ3 Mutations for Benign Familial Neonatal Convulsions Show Seizures and Neuronal Plasticity Without Synaptic Reorganization, Journal of Physiology. (2008) 586, no. 14, 3405–3423, 10.1113/jphysiol.2008.154971, 2-s2.0-48949120472. 18483067 PMC2538806

[9] Charlier C. , Singh N. A. , Ryan S. G. et al., A Pore Mutation in a Novel KQT-Like Potassium Channel Gene in an Idiopathic Epilepsy Family, Nature Genetics. (1998) 18, no. 1, 53–55, 10.1038/ng0198-53, 2-s2.0-0031974209. 9425900

[10] Reddy S. D. , Younus I. , Sridhar V. , and Reddy D. S. , Neuroimaging Biomarkers of Experimental Epileptogenesis and Refractory Epilepsy, International Journal of Molecular Sciences. (2019) 20, no. 1, 10.3390/ijms20010220, 2-s2.0-85059798973. PMC6337422 30626103

[11] Piro E. , Nardello R. , Gennaro E. et al., A Novel Mutation in KCNQ3-Related Benign Familial Neonatal Epilepsy: Electroclinical Features and Neurodevelopmental Outcome, Epileptic Disorders. (2019) 21, no. 1, 87–91, 10.1684/epd.2019.1030, 2-s2.0-85063495846. 30782577

[12] Hirose S. , Zenri F. , Akiyoshi H. et al., A Novel Mutation of KCNQ3 (c.925T > C) in a Japanese Family With Benign Familial Neonatal Convulsions, Annals of Neurology. (2000) 47, no. 6, 822–826. 10852552

[13] Liu C. , Habib T. , Salimeen M. et al., Quantification of Visible Virchow-Robin Spaces for Detecting the Functional Status of the Glymphatic System in Children With Newly Diagnosed Idiopathic Generalized Epilepsy, Seizure. (2020) 78, 12–17, 10.1016/j.seizure.2020.02.015. 32151968

[14] Bosch D. G. , Boonstra F. N. , de Leeuw N. et al., Novel Genetic Causes for Cerebral Visual Impairment, European Journal of Human Genetics. (2016) 24, no. 5, 660–665, 10.1038/ejhg.2015.186, 2-s2.0-84941213503. 26350515 PMC4930090

[15] Shevell M. I. , Sinclair D. B. , and Metrakos K. , Benign Familial Neonatal Seizures: Clinical and Electroencephalographic Characteristics, Pediatric Neurology. (1986) 2, no. 5, 272–275, 10.1016/0887-8994(86)90018-4, 2-s2.0-0022913617. 3508699

[16] Grinton B. E. , Heron S. E. , Pelekanos J. T. et al., Familial Neonatal Seizures in 36 Families: Clinical and Genetic Features Correlate With Outcome, Epilepsia. (2015) 56, no. 7, 1071–1080, 10.1111/epi.13020, 2-s2.0-84936846608. 25982755

